# Authors’ Responses to Peer Reviews of “In-hospital Mortality and the Predictive Ability of the Modified Early Warning Score in Ghana: Single-Center, Retrospective Study”

**DOI:** 10.2196/30790

**Published:** 2021-07-12

**Authors:** Enoch Joseph Abbey, Jennifer S R Mammen, Samara E Soghoian, Maureen A F Cadorette, Promise Ariyo

**Affiliations:** 1 Division of Endocrinology, Diabetes and Metabolism Department of Medicine Johns Hopkins School of Medicine Baltimore, MD United States; 2 Department of Emergency Medicine New York University Grossman School of Medicine New York University Langone Health New York, NY United States; 3 Occupational and Environmental Health Department of Environmental Health and Engineering Johns Hopkins School of Public Health Baltimore, MD United States; 4 Division of Adult Critical Care Department of Anesthesiology and Critical Care Medicine Johns Hopkins School of Medicine Baltimore, MD United States

**Keywords:** modified early warning score, MEWS, AVPU scale, Korle-Bu Teaching Hospital, KBTH, Ghana, critical care, vital signs, global health

*This is the authors’ response to peer-review reports for “In-hospital Mortality and the Predictive Ability of the Modified Early Warning Score in Ghana: Single-Center, Retrospective Study.”*

## Round 1

The authors of the manuscript [1] are grateful to the editor and reviewers [2-4] for their invaluable input and feedback.

### Reviewer AK

#### Major Comments

1. Thank you for your suggestion. We have simplified the statement of the objectives and have clarified the motivation for the study in the background, including explaining why both the modified early warning score (MEWS) and the limited MEWS (LMEWS) are included. We have revised the objectives both in the *Abstract* and in the main text. Mortality has been specified as the measured outcome of clinical deterioration and MEWS and LMEWS as the predictors. The *Methods* section has been clarified to explain the relationship between MEWS and LMEWS.
2. Thank you for your suggestion. We have modified the *Methods* section to make the statistical approach clearer to readers.
3. Thank you for your valuable suggestion. In all instances where comparisons are made, we have proceeded with MEWS followed by LMEWS, in that order.

#### Minor Comments

1. Thank you for your suggestion. We have addressed all grammatical errors.

### Reviewer BO

#### Major Comments

1. Thank you for your suggestion. We have included a power and sample size calculation in the statistical analysis (see above response to Reviewer AK [2]). Typically, patients are discharged in possession of their paper health records (electronic health records are not used, limiting study size), accounting for the smaller number of available records; we clarified this as well. However, the power calculation puts the number we were able to review in context as being 50% more than would have been needed for a significant result.
2. Thank you for drawing our attention to the lack of emphasis on the efferent arm in the study. In fact, there is no rapid response team and therefore response to deterioration is not standardized. Thus, there may be biases in the survival (eg, sicker patients getting less attention because of their perceived poor prognosis). We have now included this in the discussion of the limitations of the study.

### Reviewer CM

#### Major Comments

1. Thank you; please see the response to reviewer AK [2] as we have now included the power calculation in the *Methods* section.
2. Thank you for your observation. Missing data were only seen for the variable “organ system” and accounted for <1%. We have now included this in the *Statistical Analysis* section.
3. Thank you for your comments about blind assessment. Blind assessment of the predictors was not carried out as these are measured values retrospectively extracted from the record. Therefore, MEWS and LMEWS are not subjective—in real time when consciousness is assessed, there may be observer bias, but we did not have any such data. Since our data is randomly interpolated based on published population proportions, lack of blinding should not be an issue. We did perform a sensitivity analysis on the threshold for MEWS and LMEWS to test the published parameters in this population in case there was a source of bias that might make such cut-points variable.
4. Thank you for your observation. The maximum duration of follow-up was 32 days (included in the first paragraph of the *Methods*). We have included a flow chart of how the cohort was generated (Figure 2).
5. Thank you for your concern. The confidential nature of patient information, the protection of anonymity, and consent are paramount in record reviews; as such, ethical approval was obtained from the Institutional Review Boards (IRB) of Johns Hopkins University and the Korle-Bu Teaching Hospital (KBTH), and clearance was obtained from the Scientific and Technical Committee of the KBTH. Although reporting was anonymous, patients’ records were not, so the researchers involved in data collection and handling needed to sign a confidentiality clause. This is now captured in the *Methods* section. Data access is limited to me; I abstracted the data and ran the study analysis for a limited duration.

## Round 2

### Reviewer CM

#### Major Comments

1. Thank you for allowing us to clarify the sample size question. The study proposal submitted to the IRB required a mandatory sample size calculation. As such, this was calculated a priori based on a publication by Kyriacos et al [5]. Based on this study, a power of 80% to detect clinical deterioration in postoperative inpatients, with a significance level of .05 and a delta value of 0.45, will give us a minimum sample size of 46. A post–data collection power analysis was also performed, based on a chi-square test comparing two independent proportions. Based on the resulting analytic sample of 112 participants, with 31 in the significant MEWS category and 81 in the nonsignificant MEWS category, our study achieves a power of 95% to detect a difference in outcome percentages of at least 37% between these two groups.
2. Thank you.
3. Thank you for your suggestion. As with all retrospective study designs, the measurement of outcomes occurred prior to the start of the study; as such, we had no control over how assessments were made including choice of measurement tools, whether tools were valid and reliable, and how results were interpreted and recorded. Blinding of outcome assessors serves to limit detection bias, but this was unemployable in our retrospective chart review, and the determination of which predictors to use in our analysis is based solely on the conceptual framework described in Figure 1.
4. Thank you for your ethical concerns and the effort to maintain the highest standards in clinical research. The confidential nature of patient information, protection of anonymity, and consent are paramount in record reviews; as such, ethical approval was obtained from the IRB of Johns Hopkins University and the KBTH, as well as clearance from the Scientific and Technical Committee of the KBTH. In addition, we received a “waiver of documented (signed) permission,” which waives the requirements to obtain documented (signed) parent or guardian permission under the same conditions that apply to waiving signed consent from adult subjects. Documentation of assent and permission for adolescents 13 to 17 years of age involves being fully informed about a study and giving a signed assent to participation in a research study. They are, however, equally subject to a waiver of signed permission.
